# miR-204-5p Protects Nephrin from Enzymatic Degradation in Cultured Mouse Podocytes Treated with Nephrotoxic Serum

**DOI:** 10.3390/cells14050364

**Published:** 2025-03-01

**Authors:** George Haddad, Judith Blaine

**Affiliations:** 1 Division of Renal Disease and Hypertension, Department of Medicine, School of Medicine, University of Colorado, Aurora, CO 80045, USA; george.haddad@cuanschutz.edu; 2 Division of Renal Diseases and Hypertension, University of Colorado Anschutz Medical Campus, 12700 E 19th Ave, Aurora, CO 80045, USA

**Keywords:** podocytes, nephrin, miR-204-5p, nephrotoxic serum, lncJosd1-ps, pepstatin A, autoantibodies

## Abstract

Nephrin is an essential constituent of the slit diaphragm of the kidney filtering unit. Loss of nephrin expression leads to protein leakage into the urine, one of the hallmarks of kidney damage. Autoantibodies against nephrin have been reported in patients with minimal change disease and recurrent focal segmental glomerulosclerosis. Understanding the mechanism of nephrin loss may help improve or lead to the development of novel treatment strategies. In this study, we demonstrated the important function of miR-204-5p expression on the protection of nephrin from anti-nephrin antibodies present in nephrotoxic serum (NTS). In addition, we identified that aspartyl protease cathepsin D is one enzyme that may be involved in nephrin enzymatic degradation and that cathepsin D is a direct target of miR-204-5p gene regulation. The regulation of miR-204-5p expression was determined to be regulated by the long noncoding RNA Josd1-ps. In an NTS in vivo animal model, treatment with the pan aspartic protease inhibitor Pepstatin A ameliorated renal damage. Finally, we showed that the expression of miR-204-5p had a nephrin-protecting function in vitro. Developing a method of delivery of miR-204-5p specifically to podocytes in vivo may provide a novel method of nephroprotection against nephrin autoantibodies.

## 1. Introduction

Podocytes are key constituents of the glomerular filtration barrier (GFB), and podocyte loss leads to progressive and often irreversible decline in kidney function. The filtration barrier, which prevents the leakage of proteins and molecules larger than the size of albumin into the urine, is a complex structure dependent on the correct localization of several structural and signaling proteins [1]. Nephrin, a 150 kDa trans-membrane protein and member of the immunoglobulin superfamily, is one of the most important components of the GFB. Loss of functional nephrin in podocytes leads to significant proteinuria and irreversible renal decline [2]. Nephrin, however, can also be lost or mislocalized in acquired forms of kidney disease, particularly in immune-mediated kidney diseases [3,4]. A recent study also found the presence of anti-nephrin autoantibodies in a significant proportion of patients with minimal change disease [2]. Anti-nephrin autoantibody levels correlated with disease activity, suggesting that these autoantibodies are pathogenic. In addition, the immunization of mice with the ectodomain of murine nephrin resulted in nephrin autoantibody formation and the development of significant proteinuria together with the downregulation of proteins necessary to maintain the filtration barrier. The mechanisms underlying how nephrin autoantibodies result in nephrin degradation and damage to the filtration barrier remain unknown.

MicroRNAs (miRNAs) are 18–22 nucleotide noncoding RNAs that regulate gene expression through the binding of a “seed” sequence in the miRNA to the 3′ or, on some occasions, the 5′ UTR of a target mRNA, thereby inducing degradation or transcriptional repression of the target [5,6]. Of note is that small changes in microRNA expression can have significant effects on the target genes. Previous studies have shown that a microRNA, miR-204, which is expressed in the kidney, is protective in hypertensive and diabetic kidney disease [7]. In addition, several studies have shown that decreased expression of miR-204 exacerbates kidney injury in various animal models of renal diseases [8].

In this study, we evaluated whether miR-204 is protective in an in vitro model of podocyte stress induced by treating cultured murine podocytes with nephrotoxic serum (NTS), which contains anti-nephrin antibodies. We show that after NTS treatment, miR-204-5p overexpression protects nephrin from enzymatic degradation by cathepsin D, thereby preserving nephrin expression. We also find that lysosomal-associated membrane protein 1 (LAMP1) and cathepsin D are direct miR-204-5p targets. In addition, we demonstrate that the long noncoding RNA Josd1-ps regulates miR-204-5p function. We also show that the induction of nephrotoxic serum nephritis, an in vivo model of immune-mediated kidney disease, results in decreased kidney expression of miR-204 and that proteinuria and histologic damage can be significantly ameliorated by treatment with Pepstatin A, an inhibitor of cathepsin D. Taken together, our results suggest that after an immune-mediated insult, miR-204 protects nephrin from cathepsin D-mediated degradation.

## 2. Materials and Methods

### 2.1. Reagents

Nephrin antibody (R&D Systems Minneapolis, MN, USA, cat#AF3159, lot# CBIK0422122). Neph1 and Cathepsin E antibodies (NovusBio Centennial, CO, USA, cat# NBP3-03509, lot# 010718020, cat# NB400-152SS, lot# B-6 respectively). Podocin antibody (Invitrogen ThermoFisher, Waltham, MA, USA, cat#P A5-37904, lot# ZD4291857). LAMP1 antibody (abcam, Boston, MA, USA, cat# AB208943, lot# 1015275-34). Actin antibody (SigmaAldrich, St. Louis, MO, USA, cat# A1978, lot# 109M4849V), C5b-9 antibody (bioorbyt, Durham, NC, USA, cat# orb100655, lot# RB70). Alexa-635 phalloidin (Invitrogen ThermoFisher, Waltham, MA, USA, cat# A34054, lot# 2127282). 

### 2.2. Cell Culture

Immortalized mouse podocytes, generated from C57BL/6J mice as described in [9], were cultured from frozen stock in DMEM supplemented with 10% FBS, 50 U/mL penicillin, and 50 μg/mL streptomycin and grown at 37 °C and 5% CO_2_.

### 2.3. miRNA Transfection and NTS Treatment

MicroRNA-204-5p mimic, inhibitor, and negative control sequences were purchased from ThermoFisher (Waltham, MA, USA, mimic cat# 4464066, lot# ASO2LE3J; inhibitor cat# 4464084, lot# ASO2KMY0; control cat# 4464076, lot# ASO2JI01). The cells were transfected using HiPerFect (Qiagen, Hilden, Germany, cat# 301705, lot# 172028439), and 50 nM of each microRNA sequence—mimic, inhibitory, or control—was added to the cells dropwise and incubated for a further 72 h at 37 °C. The podocytes were then treated with nephrotoxic serum (NTS) (Probetex San Antonio, TX, USA, cat# PTX-001S-Ms, lot# 612-3T) at 1:200 dilution and incubated overnight at 37 °C and 5% CO_2_. The next day, the cells were washed with PBS, and the cell lysates were collected using RIPA buffer (50 mM Tris-HCl pH 7.4, 150 mM NaCl, 1 mM EDTA, and 1% Triton X-100) supplemented with protease inhibitor cocktail (Roche, Mannheim, Germany, cat# 04693159001).

### 2.4. SDS-PAGE and Western Blot

The cell lysate concentration was estimated using Pierce BCA protein assay, and 10 µg was resolved in 10% SDS-PAGE gel. The proteins were transferred onto a PVDF membrane and blocked with 5% skim milk in PBST. The primary antibodies were added at 1:1000 dilution prepared in blocking buffer and incubated overnight at 4 °C. The membranes were washed three times with PBST for 30 min, and anti-species secondary antibodies were added at a 1:10,000 dilution for 1 h at RT. The membranes were washed again with PBS for 30 min, developed using Immobilon Forte Western Blot Substrate (Millipore, Burlington, MA, USA, cat# WBLUF0100, lot# 221543), and exposed to an X-ray film.

### 2.5. Immunofluorescence

Mouse podocytes were seeded on 35 mm glass-bottom MatTek (Ashland, MA, USA) dishes. The cells were transfected with 50 nM of miR 204 mimic or inhibitory sequences or control sequence using HiPerFect (Qiagen, Hilden, Germany, cat# 301705, lot# 172028439) for 72 h at 37 °C and 5% CO_2_. The cells were then fixed with 4% paraformaldehyde (Electron Microscopy Sciences, Hatfield, PA, USA) and stained with nephrin and phalloidin-488. Images were obtained using Stedycon Abberior (Göttingen, Germany) and Olympus 1X81 (Tokyo, Japan) confocal microscope available at the Advanced Light Microscopy Core Facility at the Colorado University Anschutz Medical Campus. The images were deconvoluted using Huygens Essential 23.10 software (Scientific Volume Imaging, Hilversum, The Netherlands) under the conservative setting.

### 2.6. Lentivirus-Mediated LncJosd1-ps Overexpression and Antisense Oligo Knockdown

The long noncoding RNA Josd1-ps (GenBank: NG_065344.1) full sequence was cloned into the lentivirus plasmid VVPW (kind gift from Gabriele Luca Gusella, Icahn School of Medicine Mount Sinai Hospital, New York, NY, USA) and validated by sequencing. The VVPW plasmid, along with the packaging plasmid psPAX2 (Addgene, Watertown, MA, USA) and the envelope plasmid pCMV-VSV-G (Addgene), was combined with FUGENE HD (Promega, Madison, WI, USA, cat# E2311, lot# 0000553944) transfection reagent at a 3:2:1 ratio and added to ~70% confluent 293T cells. The cell supernatants were collected after 48 and 72 h post transfection and validated for LncJosd1-ps overexpression by qPCR using RNA isolated from cells infected with 20% lentivirus supernatants. The mouse podocytes were infected with LncJosd1-ps lentivirus particles in addition to 8 μg/mL of polybrene. The cells were used in experiments after 72 h of infection. LncJosd1-ps knockdown was performed using antisense oligos (ASOs). The knockdown was validated using two different ASOs at 1nM delivered using HiPerFect (Qiagen, Hilden, Germany, cat# 301705, lot# 172028439), and the experiments were performed 72 h after transfection.

### 2.7. Cytoplasmic and Nuclear RNA Extraction

The RNA from the cytoplasm and the nucleus of mouse podocytes was isolated using the NORGEN BIOTEK CORP. kit (Thorold, ON, Canada, cat# 21000, lot# 605095) as per the manufacturer’s instructions using cells grown in a monolayer protocol.

### 2.8. Quantitative PCR and microRNA Analysis

The RNA was extracted using the TRIzol (Life Technologies, Carlsbad, CA, USA, cat# 15596026, lot# 349906) method and quantified using NanoDrop 2000 (Thermo Fisher, Waltham, MA USA). One microgram of total RNA was reversed transcribed into cDNA using LunaScript (NE Biolabs, Ipswich, MA, USA, cat# E3010L, lot# 10175742). SYBR Green master mix (ThermoFisher, Waltham, MA, USA, cat# A25742, lot# 2905743) was used, and the PCR reaction was amplified using BioRad SFX96 Thermocycler. The antisense oligos (ASOs) were purchased from Integrated DNA Technologies (IDT) (Morrisville, NC, USA). Gene expression was normalized to Valosin-containing protein (VCP) and asparagine-linked protein homolog 9 (ALG9). The results are reported as log_10_ (2^−DDCt^). The primers were ordered from IDT (Coralville, IA, USA), and the primers and antisense oligos used in this study are listed in Tables S1 and S2 of the Supplementary Data. The microRNA RT-PCR was performed with the miRCURY LNA system (Qiagen Hilden, Germany), miRCURY LNA RT kit (cat# 339340, lot# 77203282), miRCURY LNA SYBR Green PCR kit (cat# 339346, lot# 77203504), and miRCURY LNA miRNA PCR Assay primers: miR-204-5p (cat# YP00206072, lot# 20304364-2) and miR-16-5p (cat# YP00205702, lot# 40501706-2).

### 2.9. Protease Inhibitor Assay

Mouse podocytes transfected with the miR-204 inhibitory sequence were loaded for 60 min at 37 °C and 5% CO_2_ with the following protease inhibitors: 0.25 mM of AEBSF (SigmaAldrich, St. Louis, MO, USA, cat# SBR00015, lot# 0000188562), 1 mM of Pepstatin A (SigmaAldrich, St. Louis, MO, USA, cat# SAE0154, lot# P318, lot# 0000144172), 20 mM of E64 (SigmaAldrich, St. Louis, MO, USA, cat# SAE0154, lot# 0000105689), and 1X protease inhibitor cocktail (Roche, Mannheim, Germany, cat# 04693159001). NTS was added at a 1:200 dilution, and the cells were incubated overnight. The next day, the cells were washed 2× with PBS, and cell lysates were collected and resolved using SDS-PAGE and Western blot.

### 2.10. Cathepsin D ELISA

Cathepsin D was detected using the mouse cathepsin D SimpleStep ELISA kit (Abcam, Waltham, MA, USA, cat# ab239420), as per the manufacturer’s instructions, and the plate was read at 450 nm using the BioTek Synergy 2 plate reader.

### 2.11. Nephrin Digestion

Nephrin digestion with cathepsin D was carried out as described previously [10]. Briefly, 10 μg of recombinant mouse nephrin (R & D Systems, Minneapolis, MN, USA, cat# 3159-NN-050, lot# NZA0422021) was incubated with 10 μg of recombinant mouse cathepsin D (R & D Systems, Minneapolis, MN, USA, cat# 1029-AS-010, lot# FVE0122021) in 100 mM of sodium citrate and 200 mM of NaCl buffer (pH 3.5) for 3 h at 37C.

### 2.12. Silver Stain

The digested nephrin was resolved on an SDS-PAGE gel and stained using silver stain (Thermo Fisher, Waltham, MA, USA, silver reagent cat# 1851130, lot# VB297147, stabilizer base reagent, cat# 1851190, lot# VC297149, and reducer aldehyde reagent, cat# 1851170, lot# VB297148) as per the manufacturer’s instructions.

### 2.13. Site-Directed Mutagenesis

The mutation of miR-204-5p binding sites in LAMP1, cathepsin D, and Josd1-ps was performed according to the New England Biolabs Q5 ^®^ site-directed mutagenesis kit (Ipswich, MA, USA, cat# E0554S, lot# 10164452) according to the manufacturer’s instructions. The primers used to insert the mutated sequence into the lentivirus plasmids were designed using the NEBaseChanger™ tool, available at the New England Biolabs website. The resulting mutant plasmids were validated by sequencing, which was performed by Quintara Biosciences (Denver, CO, USA).

### 2.14. Luciferase Assay

LAMP1, cathepsin D 3′UTR and Josd1-ps sequences were cloned into a pIS2 luciferase plasmid (a gift from David Bartel, plasmid 12177; Addgene) and validated by sequencing. Mouse podocytes were transfected with 1 ug of pIS2 plasmid using ViaFect transfection reagent (Promega, Madison, WI, USA, cat# 4981, lot# 0000514926), and the cells were transfected with 50 nM of the miR control sequence or the miR-204-5p mimic or inhibitor sequences using the transfection reagent HiPerFect (Qiagen, Hilden, Germany, cat# 301705, lot# 172028439). The luciferase activity was determined 72 h post transfection using the *Renilla* luciferase assay system (Promega, Madison, WI, USA, cat# E183A, lot# 0000537514), as described in the manufacturer’s product manual. The luciferase substrate’s background fluorescence was subtracted from all the sample readings. The luciferase assay data were normalized to protein concentrations (reporter activity/total protein).

### 2.15. Identification of miR-204 Long Noncoding RNA Binding Partners

miR-204’s interaction with long noncoding RNA was analyzed using RNA22 and miRCode prediction tools to identify potential targets with at least 6 nucleotides interacting with the miR-204-5p seed region. Other prediction tools used to predict miR-204 target genes included TargetScan and miRDB.

### 2.16. In Vivo Experiments

All animal experiments were approved by the University of Colorado’s Institutional Animal Care and Use Committee (IACUC) and performed according to the PHS Policy on the Humane Care and Use of Laboratory Animals and IACUC.

The nephrotoxic serum nephritis mouse model was performed as previously described [11]. Male C57Bl/6 mice at 8–12 weeks old were injected with 100 μL each of nephrotoxic serum purchased from Probetex Inc. (Probetex, San Antonio, TX, USA, cat# PTX-001S-Ms, lot# 612-3T) via the tail vein. The mice were euthanized after seven days. Blood and urine were collected prior to euthanasia for blood urea nitrogen (BUN) and albuminuria analysis. For the Pepstatin A experiment, following the NTS injection, Pepstatin A was administered as described previously [12]. Briefly, male C57Bl/6 mice received 20 mg/kg of Pepstatin A by intraperitoneal injection on days 1, 3, and 5 after the NTS injection, and the mice were euthanized on day 7. Control mice received 100 μL of ethanol on days 1, 3, and 5 post NTS delivery, and the mice were euthanized on day 7.

### 2.17. MTT Cell Toxicity Assay

Immortalized mouse podocytes were seeded in a 96-well tissue culture plate and grown at 37 °C and 5% CO_2_ overnight. The next day, the cells were transfected with the miR-204-5p mimic, inhibitory, or negative control sequence and treated with NTS, as described in Section 2.3. MTT (Thiazolyl Blue Tetrazolium Bromide, Biotechne, Tocris, Minneapolis, MN, USA) cat # 298-93-1, lot # 8A/261332) was added at 10 μL/well, or 5 mg/mL, and the plate was incubated for 4 h. The medium was removed, the formazan crystals were solubilized with 100 μL/well dimethyl sulfoxide (DMSO), and the plate was read at 570 nm absorbance using a BioTek Synergy 2 plate reader.

### 2.18. Statistics

The statistical analysis was performed using GraphPad Prism 10 for macOS, Version 10.4.0 (Boston, MA, USA). The datasets were analyzed using one-way ANOVA multiple comparisons to compare the mean of each column with the mean of every other column. *p* < 0.05 was considered significant.

## 3. Results

### 3.1. Nephrin Is a Target of Nephrotoxic Serum When miR-204-5p Is Inhibited

**Figure 1:**
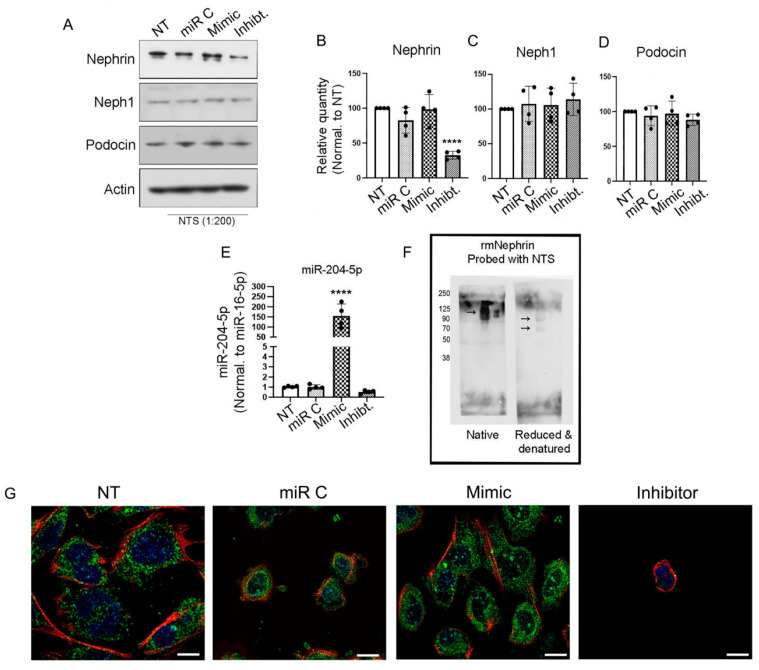
NTS targets nephrin when miR-204-5p expression is reduced. Immortalized mouse podocytes were treated with the miRNA control or miR-204-5p mimic or inhibitory sequences or left untreated prior to the addition of 1:200 nephrotoxic serum (NTS). The cell lysates were analyzed by Western blot (**A**) and probed for the podocyte’s markers nephrin, neph1, and podocin. Actin was used as a loading control. The blots are quantified in (**B**–**D**). The miR-204-5p sequence expressions were analyzed by real-time PCR, and the data were normalized to the expression of miR-16-5p (**E**). The presence of anti-nephrin antibodies in NTS was analyzed by Western blot (**F**) under native and reduced and denatured conditions. The immunofluorescence images in (**G**) show mouse podocytes transfected with miR-204-5p mimic, inhibitor, or control sequences prior to NTS treatment. The images were obtained using a Stedycon Abberior (STED) (Göttingen, Germany) and Olympus 1X81(Tokyo, Japan) confocal microscope and 100X objective lens. Nephrin is stained in green, actin is stained in red and the nucleus is stained with DAPI (blue). The scale bar represents 10 μm. The experiments were repeated 4 times (*n* = 4), and *p* values < 0.05 were considered significant, **** *p* < 0.0001.

When conditionally immortalized mouse podocytes were treated with an NTS 1:200 dilution for varying amounts of time, there were no changes in nephrin expression (Figure S1). However, when the mouse podocyte cell line was transfected with miR-204-5p mimic, inhibitory, or control sequences and then treated with a 1:200 dilution of NTS overnight (~16 h), nephrin expression changed. The cell lysates assayed by SDS-PAGE and Western blot for the podocyte markers nephrin, neph1, and podocin showed that the treatment of podocytes with the miR-204-5p inhibitory sequence and NTS resulted in a significant decrease in nephrin expression (Figure 1A) but did not affect the expression levels of neph1 and podocin (Figure 1B–D). The transfection efficiency of miR-204-5p (mimic and inhibitory) and the control sequences were analyzed by real-time PCR (Figure 1E). In addition, we demonstrate that the nephrotoxic serum contains antibodies against nephrin. Recombinant mouse nephrin was resolved on an SDS-PAGE gel and transferred to a PVDF membrane under native and/or reduced and denatured conditions. The membranes were then probed using the NTS, followed by anti-sheep IgG. As a result, nephrin was detected on the membrane under native as well as reduced and denatured gels (Figure 1F). Since the nephrotoxic serum contains an anti-nephrin antibody, we tested whether the complement system is activated. Using immunofluorescence staining, we detected some C5b-9 present in the cytoplasm of the control cells, whereas for the cells that were treated with NTS, the C5b-9 complex was observed on the cell surface as well as inside the cells (Figure S2A), suggesting that the complement system is involved in podocyte stress induced by NTS treatment. However, the complement system activation did not result in cellular death (Figure S2B). The effect of NTS on miR204 expression is shown in (G). Representative images show the expression of nephrin in mouse podocytes under normal culturing conditions (NT) and in cells treated with NTS and one of the miRNA sequences: control (miR C), miR204 mimic (mimic), and miR204 inhibitor (inhibitor).

### 3.2. Nephrin Is Degraded by an Aspartyl Protease

**Figure 2:**
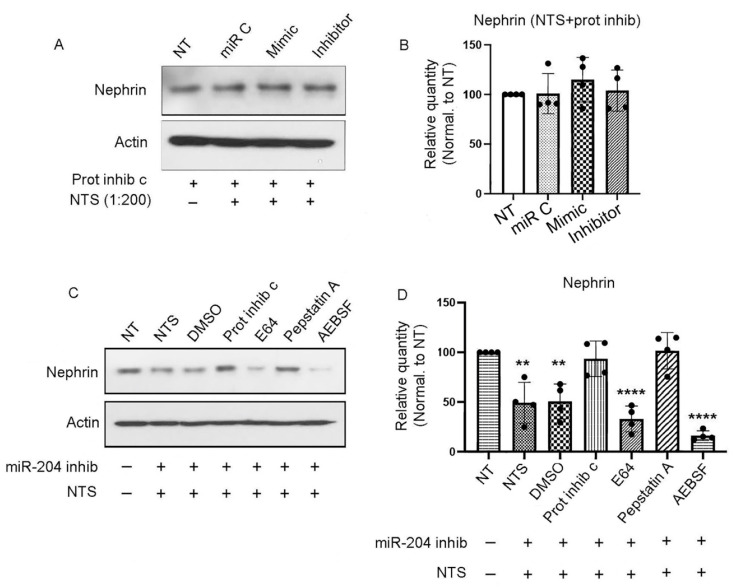
Nephrin degradation is mediated by an aspartyl enzyme. Immortalized mouse podocytes were transfected with miR-204-5p mimic, inhibitor, or control sequences and treated with protease inhibitor cocktail before treatment with NTS. As shown by the Western blot (**A**) and quantitation (**B**), protease inhibition prevented the NTS-induced degradation of nephrin, even when miR-204 was inhibited. Mouse podocytes treated with the miR-204-5p inhibitory sequence and various protease inhibitors before NTS treatment showed that the aspartic acid protease inhibitor Pepstatin A rescued nephrin from degradation (**C**), and this was evident through Western blot quantitation (**D**). The experiments were repeated 4 times (*n* = 4). *p* values < 0.05 were considered significant ** *p* < 0.003, **** p < 0.001.

To determine the nature of the enzyme responsible for nephrin degradation, podocytes were transfected with miR-204-5p mimic and/or inhibitory sequences or the control and then pretreated with 1× protease inhibitor cocktail before the addition of the nephrotoxic serum at a 1:200 dilution overnight. The next day, cell lysates were collected and assayed by SDS-PAGE and Western blot. As shown in Figure 2A,B, the decrease in nephrin expression after miR-204 inhibition in the presence of NTS was abrogated, suggesting that nephrin is degraded by an unknown protease that can be blocked by a protease inhibitor. In order to identify the enzyme responsible for nephrin degradation, we transfected mouse podocytes with an miR-204-5p inhibitor, and after 72 h, the cells were treated with various protease inhibitors, including the pan-cysteine protease inhibitor E64 (20 μM), the aspartyl protease inhibitor Pepstatin A (1 μM), the serine protease inhibitor AEBSF (250 μM), or DMSO. Thereafter, the cells were treated with nephrotoxic serum overnight, after which the cell lysates were collected and assayed by SDS-PAGE and Western blot. Treatment with E64, AEBSF, or DMSO did not rescue nephrin from degradation. However, the aspartyl protease inhibitor Pepstatin A preserved nephrin expression (Figure 2C,D), indicating that nephrin is degraded by an aspartyl protease.

### 3.3. miR-204-5p Regulates the Expression of LAMP1 and Cathepsin D

**Figure 3:**
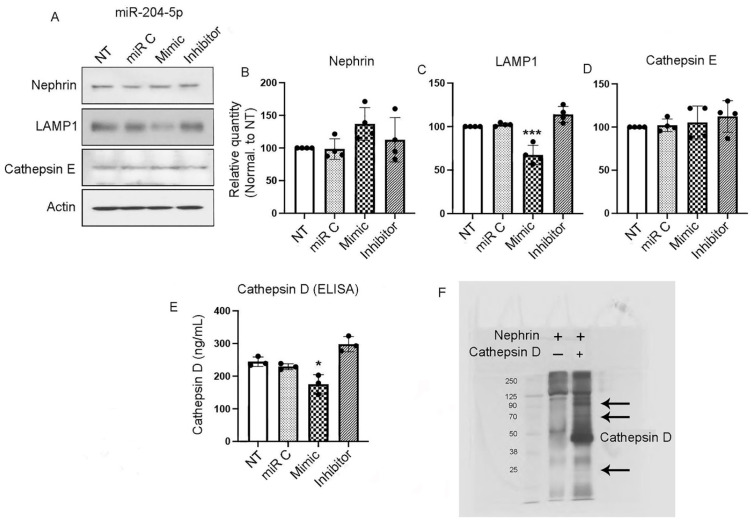
LAMP1 and cathepsin D are targets of miR-204-5p. The overexpression of the miR-204-5p mimic sequence reduced the expression of LAMP1 (**A**,**C**) but had no effect on nephrin (**A**,**B**) or cathepsin E (**A**,**D**), as determined by Western blot analysis. Using cathepsin ELISA, the overexpression of miR-204-5p decreased cathepsin D expression (**E**). The effect of cathepsin D’s enzymatic activity on nephrin is shown in (**F**) using SDS-PAGE gel and silver staining. Cathepsin D treatment results in nephrin fragments (arrows). The experiments were repeated 4 times (*n* = 4). * *p* < 0.05, *** *p* = 0.0007.

We searched in silico for candidate aspartyl proteases that could potentially degrade nephrin and could also be a direct target of miR-204-5p. Using TargetScan, we identified cathepsin D as a candidate aspartyl protease. We also identified lysosomal-associated membrane protein 1 (LAMP1) as an miR-204-5p target. We chose cathepsin E as a control since it is also an aspartyl protease but not predicted to be a target of miR-204-5p. Mouse podocytes were transfected with miR-204 mimic, inhibitory, and control sequences, and cell lysates were extracted and resolved using SDS-PAGE and Western blot. As Figure 3A,B show, the expression of nephrin was not affected in the presence of miR-204 mimic or inhibitory sequences in the absence of NTS. Interestingly, the overexpression of miR-204-5p significantly decreased the expression of LAMP1 (Figure 3A,C), indicating that LAMP1 is a target of miR-204-5p. However, the expression of cathepsin E was not affected (Figure 3A,D). Since cathepsin D expression could not be detected by Western blot, we determined the effect of miR-204-5p overexpression or inhibition on cathepsin D by ELISA. Cathepsin D expression was significantly downregulated in podocytes transfected with the miR-204-5p mimic sequence, indicating that cathepsin D is a target of miR-204-5p. The inhibition of miR-204-5p slightly increased cathepsin D expression, but this was not statistically significant (Figure 3E). In order to validate the degradation of nephrin by cathepsin D, we incubated recombinant mouse nephrin in the presence or absence of mouse cathepsin D, and the reactions were resolved on an SDS-PAGE gel and stained with silver stain. Nephrin samples incubated with cathepsin D showed several bands of nephrin fragments, indicating that cathepsin D can degrade nephrin (Figure 3F).

### 3.4. LncJosd1-ps Regulates the Expression of miR-204-5p

**Figure 4:**
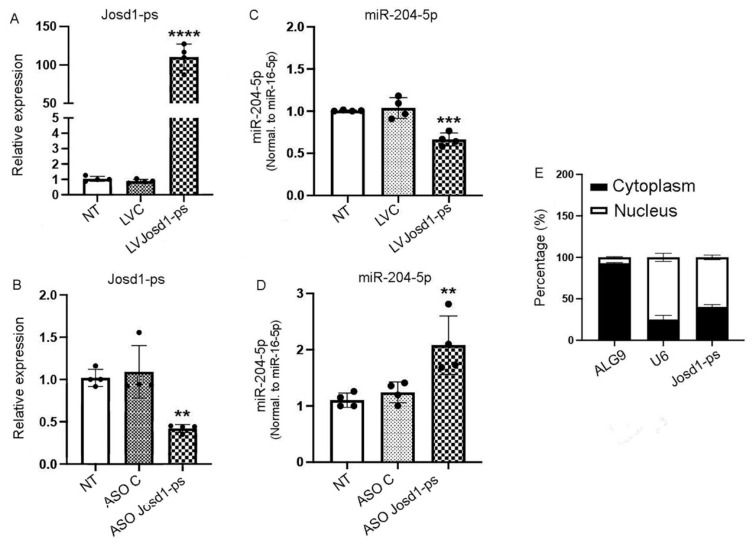
LncJosd1-ps regulates the expression of miR-204-5p. LncJosd1-ps sequence was cloned into a lentiviral vector (**A**) to overexpress lncJosd1-ps and an antisense oligo (ASO) was designed (**B**) to reduce lncJosd1-ps expression. (**C**) shows that the overexpression of lncJosd1-ps reduced miR-204-5p expression, whereas lncJosd1-ps inhibition increased miR-204-5p expression (**D**). The subcellular location of lncJosd1-ps was determined, and it shows nuclear as well as cytoplasmic expressions (**E**). The experiments were repeated 4 times (*n* = 4). *p* values < 0.05 were considered significant ** *p* < 0.003, *** *p* = 0.0002, **** *p* < 0.0001.

MicroRNAS can be regulated by long noncoding RNAs (lncRNAs), and the binding of lncRNAs to their target miRNAs can induce degradation and thus decreased expression of the target miRNA. To determine whether miR-204-5p is itself a target of lncRNAs, we screened potential candidates using several prediction tools, including DIANA, RNA22, and miRCode. Several lncRNAs were identified and screened for expression in mouse kidneys isolated from animals treated with NTS. Amongst all the screened lncRNAs, only lncJosd1-ps was upregulated when miR-204-5p expression was reduced (Figure S3). To further validate the potential lncJosd1-ps and miR-204-5p interaction, lncJosd1-ps was cloned into the lentivirus plasmid VVPW, and a virus was produced. In addition, lncJosd1-ps antisense oligos (ASOs) were designed to suppress lncJosd1-ps expression. As shown in Figure 4A,B, LV Josd1-ps induced significant overexpression of lncJosd1-ps RNA in mouse podocytes assayed 72 h post infection, whereas ASO Josd1-ps induced significant downregulation of lncJosd1-ps RNA. As expected, LV Josd1-ps significantly reduced miR-204-5p expression, whereas ASO Josd1-ps significantly increased miR-204-5p expression (Figure 4C,D). We also determined the subcellular location of lncJosd1-ps and found that ~60% of lncJosd1-ps expression is in the nucleus and ~40% is in the cytoplasm (Figure 4E).

### 3.5. Validation of miR-204-5p’s Interaction with LAMP1, Cathepsin D, and Josd1-ps

**Figure 5:**
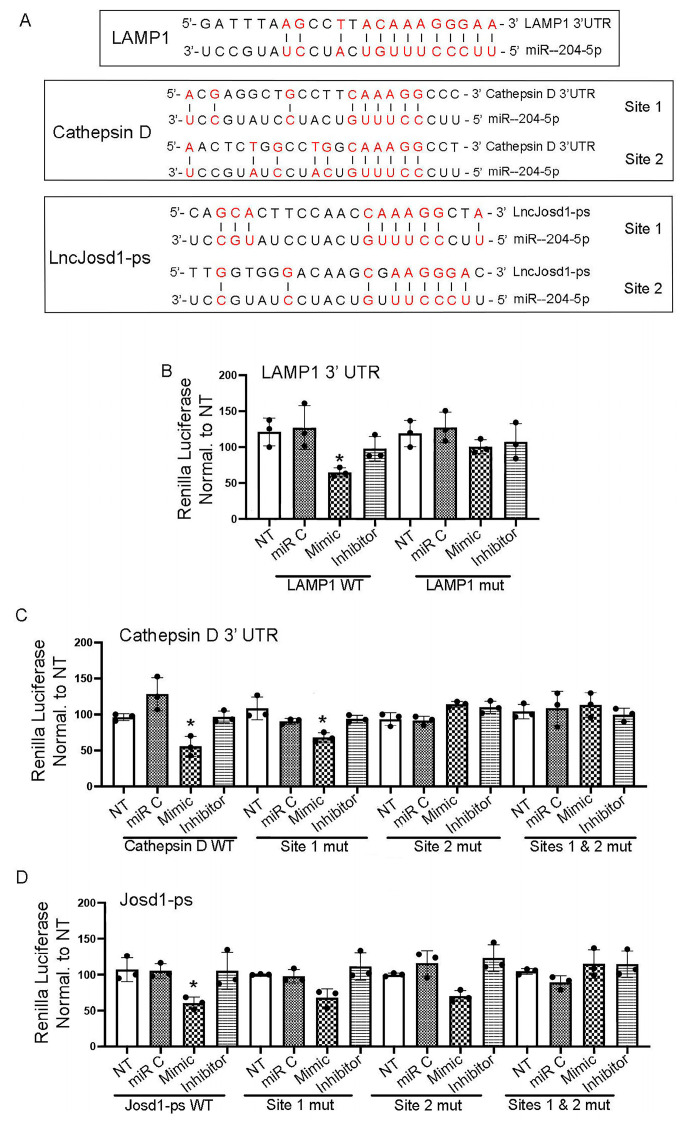
Validation of miR-204-5p targets by luciferase assay. The miR-204-5p binding sites within LAMP1 and cathepsin D 3′ UTR and within the lncJosd1-ps nucleotide sequence are shown in (**A**). miR-204-5p interaction with LAMP1 3′ UTR is validated by a luciferase assay using intact LAMP1 or mutated binding sites (**B**), cathepsin D (**C**), and lncJosd1-ps (**D**). The red letters indicate the complimentary nucleotides between miR-204-5p and the target genes sequences. The experiments were repeated 3 times (*n* = 3). * *p* value < 0.05 was considered significant from NT group.

The direct interaction between miR-204-5p and LAMP1 and cathepsin D and Josd1-ps was determined by the *Renilla* luciferase assay. The 3′ UTRs of LAMP1 and cathepsin D, as well as the entire Josd1-ps sequence, were cloned into the pIS2 luciferase plasmid. The MiR-204-5p binding site(s) was/were mutated using a Q5^®^ site-directed mutagenesis kit and specific mutant primers. Mouse podocytes were infected with a luciferase plasmid carrying an intact or mutant miR-204-5p binding site(s), followed by transfection with miR-204-5p mimic, inhibitory, or control sequences. Figure 5A shows a schematic representation of LAMP1, cathepsin D, and Josd1-ps nucleotide sequences that contain miR-204-5p binding sites. The interaction between the miR-204-5p and LAMP1 3′ UTR sequences is shown in Figure 5B. The overexpression of the miR-204-5p sequence significantly reduced luciferase activity, indicating the binding of miR-204-5p to LAMP1 3′UTR and the subsequent degradation of LAMP1. However, this effect was abrogated in cells that were infected with the LAMP1 mutant plasmid. We identified two potential miR-204-5p binding sites within the cathepsin D 3′UTR region. Luciferase analysis of this interaction is shown in Figure 5C and demonstrates that miR-204-5p binds preferably to site 2, where mutation of the nucleotide sequence of site 2 prevented miR-204-5p binding and did not affect the luciferase activity. However, the mutation of site 1 did not result in luciferase activity reduction, indicating that site 2 is the main miR-204-5p binding site within the cathepsin D 3′UTR. The double mutation of sites 1 and 2 completely abrogated miR-204-5p binding and the degradation of cathepsin D. The interaction of miR-204-5p and josd1-ps is shown in Figure 5D. Again, two potential miR-204-5p binding sites within the josd1-ps nucleotide sequence were identified. The mutation of site 1 and site 2 individually resulted in statistically insignificant reductions in luciferase activity as compared to the other treatments, indicating miR-204-5p binding to both sites. As expected, the josd1-ps double-mutant plasmid prevented miR-204-5p binding, and luciferase activity was restored to normal levels.

### 3.6. miR-204-5p Is Downregulated in Mouse Kidneys Treated with Nephrotoxic Serum

**Figure 6:**
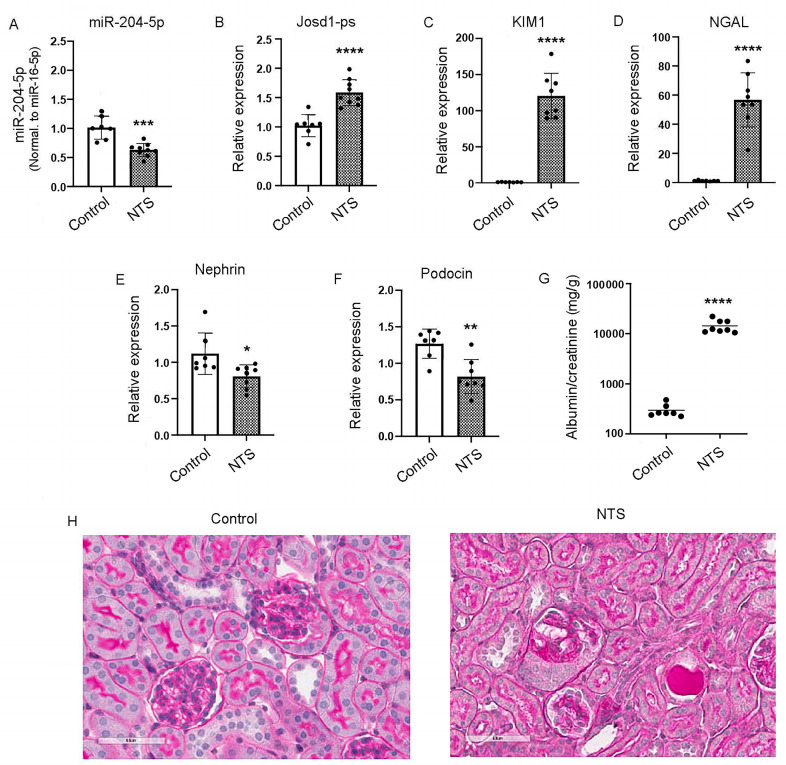
The effects of NTS on kidney function in vivo. Male C57Bl/6 mice were injected with 100 μL of NTS via the tail vein and euthanized after 1 week. Kidneys were excised, and total RNA was extracted and analyzed by real-time PCR for the expression levels of miR-204-5p (**A**), lncJosd1 (**B**), KIM1 (**C**), NGAL (**D**), nephrin (**E**) and podocin (**F**). Albuminuria was significantly increased in mice treated with NTS compared to the control mice (**G**). Figure (**H**) shows glomerular damage induced by NTS (PAS stain, scale bar 50 µm). Seven to eight animals were included per group. * *p* < 0.05, ** *p* < 0.003, *** *p* = 0.0002, **** *p* < 0.0001.

One week after treatment with nephrotoxic serum, real-time PCR showed a significant downregulation of miR-204-5p expression in mice treated with NTS as compared to the control (Figure 6A), whereas the expression level of josd1-ps was significantly increased (Figure 6B). The kidney injury markers KIM1 and NGAL were upregulated significantly in the NTS mice versus the control mice (Figure 6C,D). The podocyte markers nephrin and podocin were downregulated in the mice that received the NTS treatment compared to the control mice (Figure 6E,F). Albuminuria was also significantly increased in the NTS-treated mice compared to the control mice (Figure 6G). Histologic analysis also demonstrated increased glomerulosclerosis in the NTS-treated mice compared to the control mice (Figure 6H).

### 3.7. Pepstatin A Decreases Kidney Damage in NTS-Treated Mice by Preserving miR-204-5p Expression

**Figure 7:**
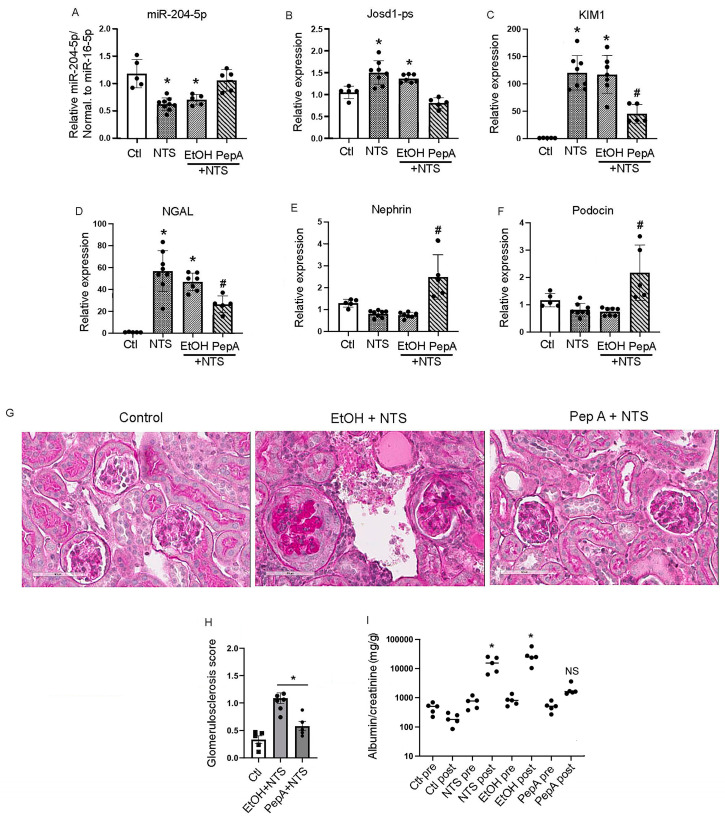
Pepstatin A reduced kidney injury in the NTS model of immune-mediated kidney disease. Mouse kidney total RNA was analyzed for miR-204-5p and lncJosd1-ps expression in animals treated with NTS followed by IP injection of Pepstatin A (20 mg/Kg) or carrier (100 µL ethanol) on days 1, 3, and 5 (**A**,**B**). The expression levels of the kidney injury markers KIM1 (**C**) and NGAL (**D**) were determined at the mRNA level along with the podocyte markers nephrin (**E**) and podocin (**F**). Kidney morphology was assessed using PAS stain (**G**). Glomerulosclerosis was determined using a 0–4 scoring system, where 0 = 0%, 1 = 25%, 2 = 50%, 3 = 75%, and 4 = 100% glomerulosclerosis. All available cortical glomeruli in a PAS-stained tissue section were analyzed (**H**). Albuminuria was determined using the albumin-to-creatinine ratio (**I**). At least 5–8 animals were used per group. * *p* < 0.05 different from control group, # different from NTS group *p* < 0.002, NS = not significant from control; scale bars: 50 µm.

The effect of Pepstatin A on miR-204-5p expression and kidney function in vivo was determined. Mice received either Pepstatin A (20 mg/kg every other day for three doses) or a vehicle alone (ethanol) post NTS injection. The analysis of mouse kidneys one week after NTS injection showed that Pepstatin A treatment protected miR-204-5p expression at a level comparable to the control mice, whereas the NTS and ethanol + NTS groups had significantly reduced levels of miR-204-5p expression (Figure 7A). In contrast, josd1-ps expression was increased in the NTS and ethanol + NTS groups, whereas Pepstatin A treatment decreased josd1-ps expression (Figure 7B). In addition, Pepstatin A treatment significantly reduced the expression of the kidney injury markers KIM1 and NGAL as compared to the mouse groups that received NTS or ethanol + NTS (Figure 7C,D). Nephrin and podocin were significantly upregulated in the mice treated with Pepstatin A prior to the administration of NTS as compared to the mice that received only NTS (Figure 7E,F). Mice pretreated with Pepstatin A had significantly lower glomerulosclerosis scores as compared to the EtOH+NTS group (Figure 7G,H), as well as a significant reduction in albuminuria compared to the mice in the NTS, EtOH, or NTS + EtOH groups (Figure 7I). Taken together, these results indicate that Pepstatin A significantly reduces kidney damage induced by NTS by protecting miR-204-5p from degradation and reducing the expression of josd1-ps.

## 4. Discussion

Podocyte injury is detrimental to proper kidney function and at the core of various glomerulopathies [2]. Identifying the different mechanisms that lead to podocyte injury aids in the development of novel therapeutic measures to treat podocytopathies.

Several podocyte antigens, such as PLA2R1, THSD7A, NELL1, HTRA1, and nephrin, are the primary targets of deleterious autoantibodies in glomerulonephritis patients [13,14,15,16,17]. Of all those antigens, nephrin is an integral component of the slit diaphragm and is directly involved in preventing large proteins from leaking into the urine [18]. Various in vivo studies have demonstrated the importance of miR-204 expression for the preservation of kidney function. The reported mechanisms involve the decreased expression of certain long noncoding RNAs that block miR-204 activity, such as MALAT1 [19] and Kcnq1ot1 [20], or the activation/deactivation of signaling pathways, including HMX1 [21], Smad5 [22], NLRP3 inflammasome [20], and Fas/FasL [8]. However, a direct link between miR-204 function and podocytes has not been reported. In this study, we describe the mechanisms underlying the miR-204-5p protection of nephrin after NTS-induced stress.

The nephrotoxic serum model is widely used as an in vivo model of acute and chronic glomerulonephritis [23]. However, the specific effect of NTS on podocytes in vitro is not clear. Given the reported function of miR-204 in protecting kidney function, we sought to explore the effect of miR-204 modulation on podocytes in the presence of NTS. Initial experiments with an NTS time course in a mouse podocyte cell line did not show a change in nephrin expression. However, when podocytes were transfected with the miR-204-5p inhibitory sequence and treated with NTS, nephrin expression was significantly decreased, whereas the levels of neph1 and podocin remained unchanged. This result suggests that miR-204-5p protects nephrin from the toxic effects of NTS. Interestingly, when podocytes were grown on a glass cover slip for immunofluorescence staining, the effect of the miR-204-5p inhibitory sequence and NTS was so drastic that the majority of cells did not survive and the remaining cells were almost devoid of nephrin. In contrast, the presence of miR-204-5p was protective even at a low level of expression in the cells that received the microRNA control sequence, and in the cells that received the miR-204-5p mimic sequence, there was a strong protective effect on nephrin expression and the overall viability of the cells. Since the NTS contains anti-nephrin antibodies, this suggests that the complement system might be activated. Immunofluorescence staining of the control and NTS-treated cells showed the intracellular location of C5b-9 in the control cells; however, the NTS-treated cells displayed surface and cytoplasmic C5b-9 locations. Notwithstanding the complement activation, there was no detectable cell lysis even though C5b-9 deposition on the cell surface was observed. It is not surprising that podocytes can resist complement-mediated cell killing. Previous reports have also shown that in vitro-cultured podocytes can withstand complement activation in a mechanism that involves several complement regulatory molecules, such as factor H, CD46, CD55, and CD59, that the podocytes possess [24,25].

The loss of nephrin in podocytes treated with NTS and the miR-204-5p inhibitory sequence can be attributed to enzymatic activity, as we observed a restoration of nephrin expression when the cells were pretreated with a protease inhibitor cocktail prior to treatment with NTS and the miR-204-5p inhibitory sequence. In the search for a potential enzyme group that is responsible for nephrin degradation, we pretreated the podocytes with various enzyme inhibitors, including the cysteine protease inhibitor E64, the aspartyl enzyme inhibitor Pepstatin A, and the serine protease inhibitor AEBSF. Nephrin was only rescued by Pepstatin A. The results suggested the involvement of an aspartic enzyme(s) and a group of proteases that includes pepsins, renins, and cathepsins [26]. Amongst this group of proteases, the cathepsins seemed more likely candidates to be involved in nephrin degradation. By screening the 3′ UTR of the two cathepsin aspartic proteases cathepsin D and E, we identified cathepsin D as a potential target of miR-204-5p, which was validated using a luciferase assay. The interaction between cathepsin D and nephrin was demonstrated by the cleavage of recombinant mouse nephrin by mouse cathepsin D. This result suggested that in the absence of miR-204-5p expression, cathepsin D, under cellular stress, such as that incurred by NTS, can act upon nephrin to reduce its expression. It is interesting to note that cathepsin D gene expression has been found to be significantly upregulated in human glomeruli in FSGS [27]. In addition, Hodgin et al. have found that the expression of CCDC91, a trans-Golgi network accessory protein that is important for sorting cathepsin D for the lysosome, is significantly negatively correlated with FSGS [28]. In diabetic nephropathy, nephrin is internalized inside endocytic vesicles mediated by dynein light chain 1 (*DYNLL1*), and eventually, nephrin is degraded in the lysosome. In the absence of *DYNLL1* expression, endocytosed nephrin was recirculated back to the plasma membrane [29]. Another mechanism of nephrin degradation may also involve the SEL1L-HRD1 protein complex for endoplasmic reticulum-associated degradation (ERAD), which is highly expressed in podocytes where nephrin is an endogenous substrate [30]. However, this degradation pathway was not investigated in this study.

The expression level of mir-204-5p was downregulated in mice treated with NTS, suggesting that a long noncoding RNA may regulate miR-204-5p activity. Several lncRNAs reportedly regulate miR-204-5p function, including MALAT1, KCNQ1OT, and SPANXA1-OT1 [19,20,22], along with other potential in silico-identified LncRNA partners of miR-204-5p, which were assayed for expression in the mice treated with NTS. Amongst all the assayed lncRNAs, only LncJosd1-ps was upregulated, suggesting a potential interaction with miR-204-5p that merited further validation. No reports were found that described a function for lncJosd1-ps. We cloned lncJosd1 into a lentivirus vector and designed antisense oligos in order to manipulate its expression and determine whether it has any effect on miR-204-5p expression. The results clearly showed a link between lncJosd1-ps expression and that of miR-204-5p, suggesting a potential binding partnership. Indeed, lncJosd1-ps overexpression led to miR-204-5p suppression, and lncJosd1-ps inhibition increased miR-204-5p expression. This binding partnership was further validated and confirmed using a luciferase assay. The cellular location of lncJosd1-ps is important for its “sponging” of microRNA function. Although around 60% of lncJosd1-ps transcripts are present in the nucleus, the remaining 40% are available in the cytoplasm for potential sponging activity.

There is a general agreement in the scientific literature that the expression of miR-204 is protective in various kidney injury models [7,8,19,20,21,22,31]. However, the nephroprotective function of miR-204 has never previously been linked to nephrin. Here, we show a correlation between miR-204 and nephrin expression and ultimately kidney function. In the NTS animal model, miR-204-5p expression was significantly downregulated, which coincided with nephrin’s reduced expression. As a result, the renal injury was severe, as measured by the elevated expressions of the kidney injury markers KIM1 and NGAL as well as the albumin/creatinine ratio with noticeable glomerular injury. In contrast, Pepstatin A treatment in vivo protected miR-204-5p and nephrin expression and overall kidney function. Inhibiting aspartyl proteases had a beneficial effect on kidney function after NTS treatment, but other potential effects of inhibiting aspartyl proteases remain to be determined. Additional limitations of this study include the fact that the NTS model is not an ideal model of anti-podocyte antigen-mediated glomerular disease as NTS, besides containing anti-nephrin antibodies, contains a variety of other antibodies against glomerular antigens. Additionally, the studies demonstrating directly that the upregulation of miR-204-5p directly protects podocyte nephrin from degradation were all performed in cultured podocytes in vitro. However, such detailed mechanistic studies are impossible to perform in podocytes in vivo given the cytoarchitecture and location of podocytes in vivo.

It has proven very challenging to deliver microRNAs effectively to podocytes. However, if miRNAs can be delivered to podocytes with high efficiency, miR-204 remains an excellent candidate for potential renal therapy for glomerular diseases in which nephrin is degraded. Our findings are of special relevance given recent findings that anti-nephrin autoantibodies are present in a significant number of patients with minimal change disease [4]. However, the mechanisms underlying how these anti-nephrin autoantibodies lead to loss of nephrin from the slit diaphragm remain to be determined.

## 5. Conclusions

The data presented in this study showed the importance of miR-204-5p expression in protecting nephrin from enzymatic degradation by the aspartic enzyme cathepsin D under stress conditions caused by nephrotoxic serum and miR-204-5p inhibition. Mechanistically, we showed that when miR-204-5p is present, it interferes with nephrin degradation inside the lysosome by regulating the expressions of the lysosome proteins cathepsin D and LAMP1. In the event of podocyte stress, the long noncoding RNA Josd1-ps is upregulated and miR-204-5p is “sponged up”, which in turn renders the internalized nephrin/ab immune complex susceptible to enzymatic degradation inside the lysosome. Therefore, the interplay between miR-204-5p, LncJosd1-ps and cathepsin D determines whether internalized nephrin is rescued from enzymatic degradation or not. Our results suggest that miR-204-5p is nephroprotective, and devising a strategy to deliver this micro-RNA specifically to podocytes could have therapeutic potential in the future.

## Data Availability

All data will be made available to all interested scientists upon request.

## Supplementary Materials

The following supporting information can be downloaded at https://www.mdpi.com/article/10.3390/cells14050364/s1: Table S1: A list of primers; Table S2: A list of antisense oligos. Figure S1: The effect of NTS on mouse podocytes. Figure S2: The complement system is activated but podocytes survive the complement attack. Figure S3: The expression of various lncRNAs in mouse kidneys treated with NTS.
