# Supplementary material for: miR-204-5p Protects Nephrin from Enzymatic Degradation in Cultured Mouse Podocytes Treated with Nephrotoxic Serum
Source: Cells. 2025 Mar 1;14(5):364. doi: 10.3390/cells14050364 (PMC11899291; doi:10.3390/cells14050364)
Supplement: Supplementary file 1 [file cells-14-00364-s001.zip › cells-3475557-supplementary.pdf]

Supplementary Materials

Table S1: A list of primers

	Sense	Antisense
ALG9	5-GAAGCACAGGTGGAAGAGTT-3’	5’-AACCACCAACTTCCCGTAATAG-3’
Podocin	5’-GCCTCTCTTCTTCTAAGCAGT-3’	5’-TTTCAGTGAGGGATCGATGTG-3’
Nephrin	5’-TTCAGACCACACCAACATCC-3’	5’-AGGTTTCCACTCCAGTCCTA-3’
NGAL	5’-ACCAGTTCGCCATGGTATTT-3’	5’GGGTGAAACGTTCCTTCAGT-3’
KIM1	5’-CTCCAAGAAGACCCACAACTAC-3’	5’-GGAGGTAGAGACTCTGGTTGAT-3’
LncJosd1-ps	5’-AAGAAGTGGAAGCCCATCAG-3’	5’-AGGCCACAGCACATCAAA-3’
LncGM11633	5’-GCTTGCTGGGTGACATACTT-3’	5’-GGGCTCAGAAACCAGATCATAG-3’
LncDnm3os	5’-GGCTATTTGCATAGTCCTACCC-3’	5’-TTCCTGCCCTGGAAGAATAAC-3’
LncMir100hg	5’-TCCCTGCCACCACAAATAC-3’	5’-GCTCGGTTAGCTACCTTCATAG-3’
LncOip5os1	5’-GCTTTGCTGTTCTAGTCCTTCT-3’	5’-CAGTTCAGGGTGAGCATTGT-3’
LncDleu2	5’-GCTGTGCTCTCCTTCGTAAT-3’	5’-GAAACACTGTGGTGCAAACC-3’
LncRiken5031425E22	5’-GCAGTACTCGGATACACTGAAC-3’	5’-CCAAGTCAGAGAGGCTAGAGA-3’
LncGm17936	5’-CCAGGCTCTGTGAAGAAAGT-3’	5’-CTTCATACCGTAAGCCTGGATAG-3’
snRNA U6	5’-GGCCACCCACATCTACATAC-3’	5’-ACCAGTCTCGGTACAAACATC-3’

Table S2: A list of antisense oligos

LncJosd1-ps ASO1	5’-mA*mG*mA*mG*mC*T*C*C*C*T*G*C*G*C*T*mG*mC*mU*mU*mC-3’
LncJosd1-ps ASO2	5’-mG*mA*mG*mA*mG*G*C*A*A*C*T*T*G*A*G*mU*mG*mG*mA*mC- 3’

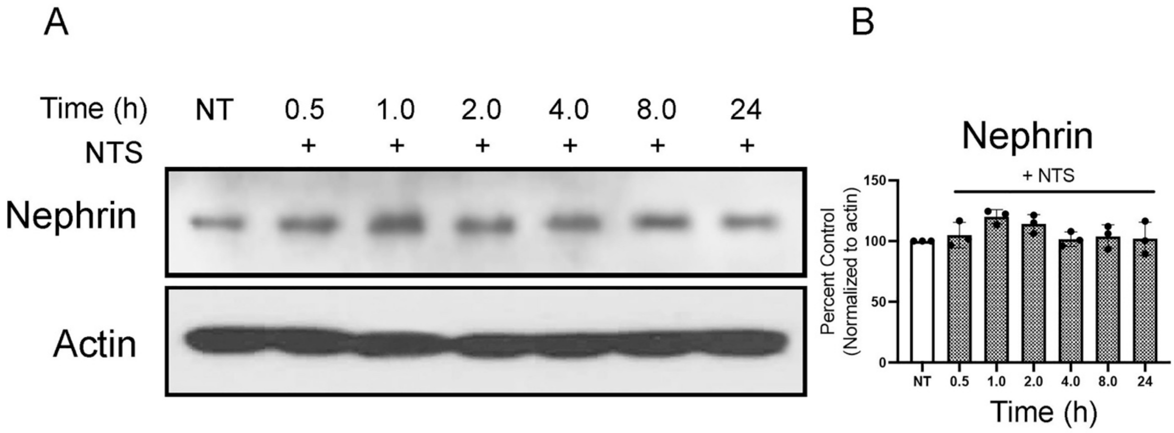

Figure S1: The effect of NTS on mouse podocytes.

Immortalized mouse podocytes were treated with 1:200 dilution of NTS over the indicated time points. The cell lysates were collected and resolved over SDS-PAGE gel and transferred to a PVDF membrane. The membrane was probed for nephrin and then stripped, blocked and re-probed for actin (A). The bands densitometry was analyzed and the results were normalized to actin. The bar represents the SEM of three independent experiments performed.

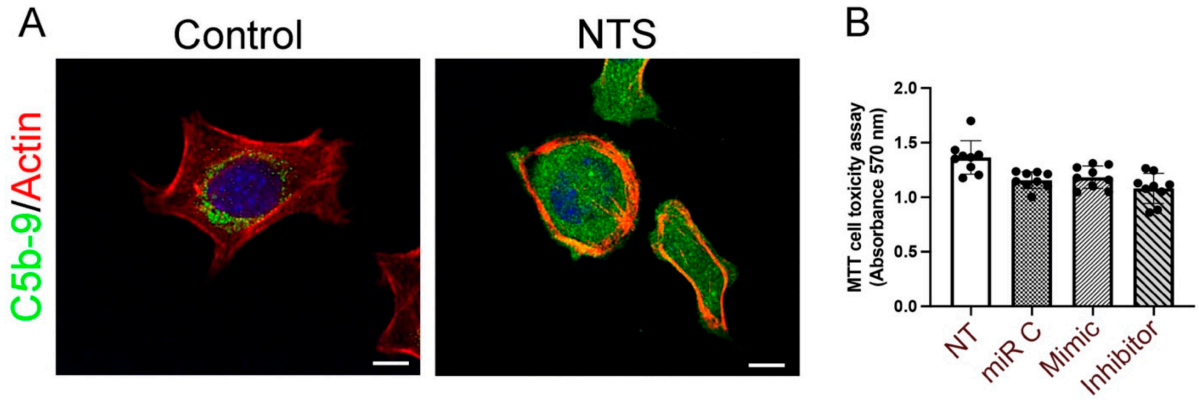

Figure S2: The complement system is activated but podocytes survive the complement attack

Mouse podocytes were grown on glass cover slips and treated with 1:200 NTS supplemented with 1 % mouse serum and incubated overnight at 37C and 5 % CO_2. The cells were fixed and double stained with anti-mouse C5b-9 and phalloidin-635. The images were captured using confocal microscope and 100 X objective lens. The bar represents 10 µm (A). MTT cell toxicity assay was performed to determine the survivability of the mouse podocytes subjected to microRNA transfection and NTS treatment (B). The bar represents the SEM of three independent experiments ran in triplicates.

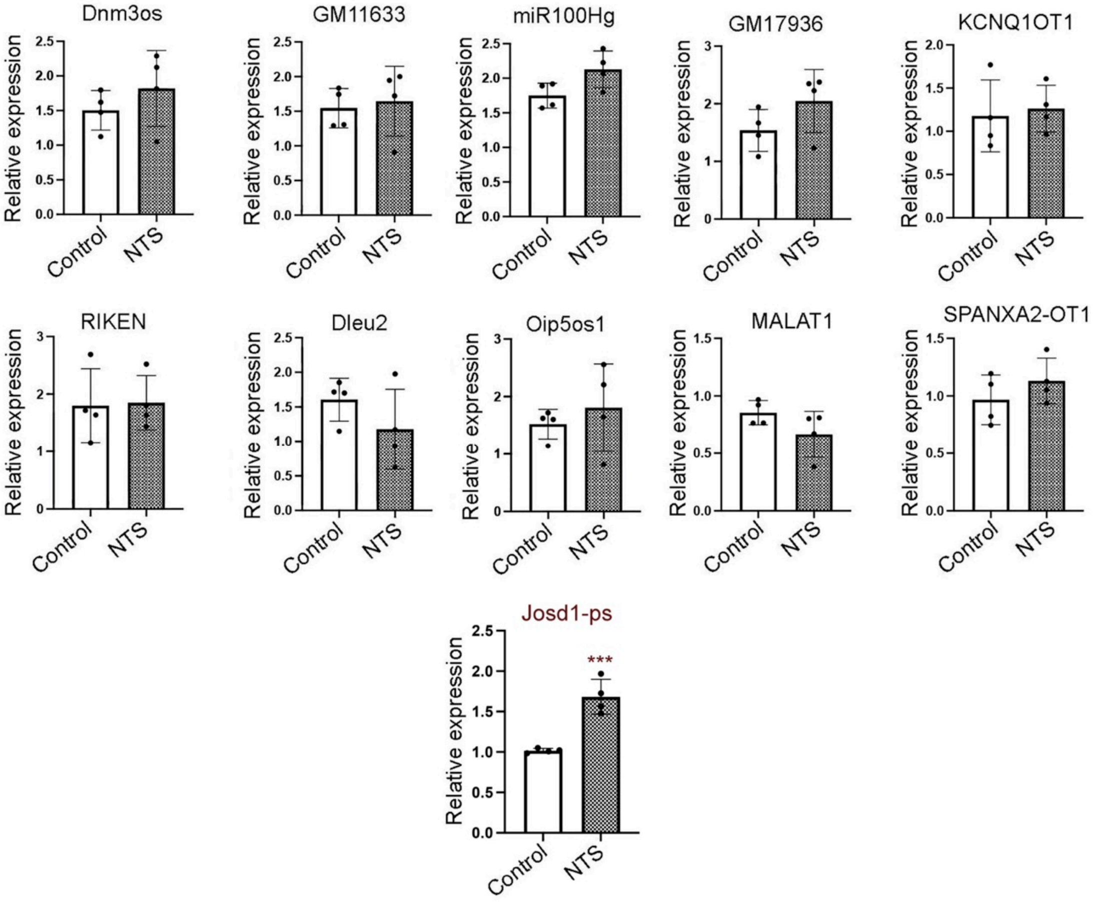

Figure S3: The expression of various lncRNAs in mice kidneys treated with NTS.

Several lncRNAs that contain a miR-204-5p binding site were screened by real-time PCR in search of a possible miR-204-5p binding partner(s) in male mice treated with NTS or control nonspecific IgG. The bar represents the SEM of four mice included in each group. *** *P* = 0.0009
